# Supplementary material for: End Sequence Analysis Toolkit (ESAT) expands the extractable information from single-cell RNA-seq data
Source: Genome Res. 2016 Oct;26(10):1397–410. doi: 10.1101/gr.207902.116 (PMC5052061; doi:10.1101/gr.207902.116)
Supplement: Supplemental Material [file supp_gr.207902.116_Supp_Materials.tar.gz › ESAT-esat_v0.1/doc/html/classbroad_1_1core_1_1datastructures_1_1_j_c_s_cache.html]

ESAT: broad.core.datastructures.JCSCache Class Reference


ESAT


- Main Page
- Related Pages
- Packages
- Classes
- Files

- Class List
- Class Index
- Class Hierarchy
- Class Members

All Classes Namespaces Files Functions Variables Enumerator Pages

- broad
- core
- datastructures
- JCSCache

Classes |
Public Member Functions |
Static Public Member Functions |
List of all members

broad.core.datastructures.JCSCache Class Reference

Collaboration diagram for broad.core.datastructures.JCSCache:

[legend]

|  |  |
| --- | --- |
| Classes | |
| class | JCSNodeIterator |
|  | |

|  |  |
| --- | --- |
| Public Member Functions | |
|  | JCSCache (PairedEndReader reader, int cacheSize, JCSAlignmentModel m) |
|  | |
| FilteredIterator | query (Annotation window, boolean fullyContained, CoordinateSpace cs) |
|  | |
| CloseableIterator< AlignmentCount > | getReads (Annotation w, boolean fullyContained) |
|  | |
| CloseableIterator< AlignmentCount > | getReads () |
|  | |

|  |  |
| --- | --- |
| Static Public Member Functions | |
| static Integer | generateRandom (int length) |
|  | |
| static void | dispose (String cacheName) |
|  | |

## Constructor & Destructor Documentation

|  |  |  |  |
| --- | --- | --- | --- |
| broad.core.datastructures.JCSCache.JCSCache | ( | PairedEndReader | *reader*, |
|  |  | int | *cacheSize*, |
|  |  | JCSAlignmentModel | *m* |
|  | ) |  |  |

Initializes the cache

Parameters
:   |  |  |
    | --- | --- |
    | reader |  |

## Member Function Documentation

|  |  |  |  |  |  |
| --- | --- | --- | --- | --- | --- |
| static void broad.core.datastructures.JCSCache.dispose | ( | String | *cacheName* | ) |  |

static

Shuts down the specified cache, and removes it from the list of active caches so it can be created again.

Parameters
:   |  |  |
    | --- | --- |
    | cacheName | The name of the cache to dispose of. |

|  |  |  |  |  |  |
| --- | --- | --- | --- | --- | --- |
| static Integer broad.core.datastructures.JCSCache.generateRandom | ( | int | *length* | ) |  |

static

Generates a Long random number of length "length". We will use 10 digits

Parameters
:   |  |  |
    | --- | --- |
    | length |  |

Returns

|  |  |  |  |
| --- | --- | --- | --- |
| CloseableIterator<AlignmentCount> broad.core.datastructures.JCSCache.getReads | ( | Annotation | *w*, |
|  |  | boolean | *fullyContained* |
|  | ) |  |  |

Here is the caller graph for this function:

|  |  |  |  |  |
| --- | --- | --- | --- | --- |
| CloseableIterator<AlignmentCount> broad.core.datastructures.JCSCache.getReads | ( |  | ) |  |

|  |  |  |  |
| --- | --- | --- | --- |
| FilteredIterator broad.core.datastructures.JCSCache.query | ( | Annotation | *window*, |
|  |  | boolean | *fullyContained*, |
|  |  | CoordinateSpace | *cs* |
|  | ) |  |  |

---

The documentation for this class was generated from the following file:

- java/broad/core/datastructures/JCSCache.java


---

Generated on Fri Aug 15 2014 15:10:51 for ESAT by  

 1.8.7
